# Identification of novel biomarkers of acute phase response in chickens challenged with *Escherichia coli* lipopolysaccharide endotoxin

**DOI:** 10.1186/s12917-024-04062-3

**Published:** 2024-06-01

**Authors:** Francesca Riva, Peter D. Eckersall, Christopher C. Chadwick, Laura C. Chadwick, Dorothy E. F. McKeegan, Jorge Peinado-Izaguerri, Geert Bruggeman, David Hermans, Mark McLaughlin, Maureen Bain

**Affiliations:** 1 School of Health and Life Sciences, University of the West of Scotland, High St, Paisley, PA1 2BE UK; 2 School of Biodiversity, One Health and Veterinary Medicine, University of Glasgow, Bearsden Rd, Glasgow, G61 1QH UK; 3 Faculty of Veterinary Medicine, University of Zagreb, Radoslava Cimermana, Zagreb, 10000 Croatia; 4 Life Diagnostics, P124 Turner Lane, West Chester, PA 19380 USA; 5 Department of Microbiology and Immunology, University of Veterinary Medicine and Pharmacy in Košice, Komenského, Košice 041 81 Slovakia; 6 Nutrition Sciences N. V, Booiebos, Ghent B-9031 Belgium; 7 Division of Molecular and Cellular Function, School of Biological Sciences, University of Manchester, Manchester, M13 9PT UK

**Keywords:** Broiler chicken, Acute phase proteins, Lipopolysaccharide endotoxin, Serum amyloid A, Alpha-1-acid-glycoprotein, Hemopexin, Extracellular fatty acid binding protein, Plant extracts

## Abstract

**Background:**

The chicken’s inflammatory response is an essential part of the bird’s response to infection. A single dose of *Escherichia coli* (*E. coli*) lipopolysaccharide (LPS) endotoxin can activate the acute phase response (APR) and lead to the production of acute phase proteins (APPs). In this study, the responses of established chicken APPs, Serum amyloid A (SAA) and Alpha-1-acid-glycoprotein (AGP), were compared to two novel APPs, Hemopexin (Hpx) and Extracellular fatty acid binding protein (Ex-FABP), in 15-day old broilers over a time course of 48 h post *E.coli* LPS challenge. We aimed to investigate and validate their role as biomarkers of an APR. Novel plant extracts, Citrus (CTS) and cucumber (CMB), were used as dietary supplements to investigate their ability to reduce the inflammatory response initiated by the endotoxin.

**Results:**

A significant increase of established (SAA, AGP) and novel (Ex-FABP, Hpx) APPs was detected post *E.coli* LPS challenge. Extracellular fatty acid binding protein (Ex-FABP) showed a similar early response to SAA post LPS challenge by increasing ~ 20-fold at 12 h post challenge (*P* < 0.001). Hemopexin (Hpx) showed a later response by increasing ∼5-fold at 24 h post challenge (*P* < 0.001) with a similar trend to AGP. No differences in APP responses were identified between diets (CTS and CMB) using any of the established or novel biomarkers.

**Conclusions:**

Hpx and Ex-FABP were confirmed as potential biomarkers of APR in broilers when using an *E. coli* LPS model along with SAA and AGP. However, no clear advantage for using either of dietary supplements to modulate the APR was identified at the dosage used.

## Background

The broiler innate immune system is highly complex and serves as the front line of the host’s defence [1]. Components that originate from gut microbiota, such as lipopolysaccharides (LPS), lipoteichoic acid, peptidoglycan, flagellin and bacterial DNA, can stimulate the innate immune response [2, 3]. These components can be found on the outer membrane of Gram-negative bacteria such as *Escherichia coli (E. coli)* [4]. Researchers often use *E. coli* LPS in challenge experiments to evaluate an animal’s ability to respond to an inflammatory stimulus [4]. The immune response of the young broilers to a single dose of LPS leads to the production of inflammatory molecules such as cytokines and chemokines [5], that can initiate a complex network of secondary reactions including the acute phase response (APR) [6]. The characterization of APR in chickens following an LPS challenge has been well defined by the manifestations of fever, anorexia, inflammation and the production of acute phase proteins (APPs) [6–8]. APPs are predominantly produced in the liver and then secreted into the blood. Thus, the presence of increased APPs in the plasma serves as a useful physiological biomarker to characterize a disease challenge [9]. Among the most established APPs in broilers, Serum amyloid A (SAA) and Alpha-1-acid-glycoprotein (AGP) have been shown to increase more than 100-fold and 5-fold respectively following an LPS challenge [10, 11]. Recently, two new potential biomarkers of LPS induced APR, Hemopexin (Hpx) and extracellular fatty acid binding protein (Ex-FABP) precursor have been identified in broilers of 15 days old, using a proteomics approach [12]. However, the value of using these proteins as biomarkers of infection in chickens needs to be further assessed as they have not been validated by the use of an independent assay system [12] and no further information on their role as APPs is available from the literature. The availability of immunoassays for chicken Hpx and Ex-FABP means that they can now be compared in their APR to more established chicken APP.

Furthermore, a variety of feed additives have been proposed to enhance the hosts’ ability to cope with the the inflammatory response initiated by LPS as well as promote the broiler’s growth performance [13]. Novel plant extracts, such as citrus (CTS) and cucumber (CMB) extracts, have been identified as potentially effective anti-inflammatory and antimicrobial growth promoter candidates in broiler diets due to the potential beneficial effects of their bioactive compounds, widespread availability and low purification costs [14–17]. Citrus extract, for example, is particularly rich in pectin (a source of soluble dietary fibre), polyphenols (including flavonoids), carotenoids, and essential oils (including Limonene) [18]. The beneficial effects of dietary fibres are mainly attributed to their fermentation products which produce anti-inflammatory short-chain fatty acids (SCFAs) [19]. Polyphenols, carotenoids and limonene have been shown to produce positive effects on the immune system thanks to their antioxidant and anti-inflammatory properties [20–22]. Similarly, the presence of vitamins, β-carotene and polyphenols in cucumber (CMB) extracts make it another candidate for modulating the immune system of broilers [23, 24]. However, the mechanism of action and interaction between the host and molecules provided through the supplementation need to be clarified [25–27]. To the best of our knowledge, there is no full experimental information on whether the APR in young broilers can be attenuated by dietary supplementation with novel plant extracts such as CTS and CMB. In this study, an *E.coli* LPS challenge was used to compare the APR in young broilers (15-days old) fed CTS, CMB and unsupplemented control (CTL) diet by measuring the plasma concentrations of SAA, AGP, Hpx and Ex-FABP. The aims of this study were to investigate the role of Hpx and Ex-FABP as novel APR biomarkers as well as to evaluate the anti-inflammatory activity of CTS or CMB dietary supplements in challenged broilers.

## Methods

### In vivo trial and blood sampling

A total of 144 one-day-old male broiler chickens (Ross 308) were obtained from a commercial hatchery (PD Hook Hatcheries Ltd, Bampton, UK) and raised for 17 days at Cochno Farm & Research Centre, Glasgow [28]. At day 0, each bird was tagged with a uniquely numbered, weighed and randomly allotted to one of three diets: starter diet without any supplements (CTL), starter diet with citrus extract supplement (CTS) (300 g/ton diet), starter diet with cucumber extract supplement (CMB) (75 g/ton diet). The chicken starter (day 0 to 14) and grower (day 14 to 17) corn-soybean meal-based diets were formulated and prepared at NuScience, Ghent, Belgium (Table 1). Broilers were provided ad libitum access to water and feed throughout the trial. Each experimental dietary group consisted of 12 chickens randomly allocated to 12 pens (4 replicate pens/each diet) of 2.5 m^2^ size on a litter of wood shavings. Each pen was equipped with a spot brooder, feeder, drinker and litter. Room temperature was set at 35 °C at the start of the experiment and gradually reduced of 1 °C each three days until 20 °C at day 28 with humidity > 50%, as recommended in the Ross 308 breeder management guide (Aviagen, Midlothian, UK). Lighting started with 23-h light and 1 h darkness (23 L:1D) from day 1 to day 7 and gradually decreased to 18 L:6D on day 17.

At the age of 15 days old, blood was collected from 12 birds per diet (3 birds per pen and replicate) (T0h). Birds were then challenged with a single dose of *E.coli* LPS (LPS from *E. coli* O111:B4 purified by phenol extraction, L2630-25MG; Sigma-Aldrich, Dorset, UK) (2 mg/kg body weight) subcutaneously injected in a volume of 0.5 ml of sterile saline. Blood was collected at 12 (T12h), 24 (T24h) and 48 (T48h) hours post LPS injection, from the same number of birds per diet (*N* = 12). The study of [29] provided the baseline data to monitor the APP response of the birds used in this experiment. During the trial, one bird on the CTL diet died following the LPS challenge reducing the sample size to 11 in this group. At each time point approximately 1 ml of blood was taken by venepuncture of the brachial vein and placed in heparinized tubes. The blood samples were then centrifuged (2000 x g) for 15 min at 4^◦^C and the plasma aliquots immediately frozen at -20^◦^C. After T48h, the trial was ended and all birds were humanely euthanised by Schedule 1 procedure by administering an overdose of a Barbiturate anaesthetic agent (1 ml/kg of Pentobarbital sodium R Euthatal Dopharma Research B.V.), injected into the brachial vein.

**Table 1:** Basal starter and grower diet used in the in vivo trial

Feed ingredients	Starter diet (g/100kg) (0 to 14 days)	Grower diet (g/100kg) (14 to 17 days)
Corn	25.000	25.000
DL-methionine	0.107	0.081
L-Lysine HCI	0.234	0.266
L-Threonine	0.097	0.105
Premix Minevita Bro	3.000	3.000
Monteban 100	0.060	0.060
Sodium Bicarbonate	0.196	0.089
Soya bean meal 47%CP + 2%CP	27.450	22.114
Soya bean oil refined	1.880	3.072
Soya beans Danex	7.500	7.500
Vit Choline Chloride 60% Veg.	0.010	0.010
Xylanase	0.010	0.010
Wheat enzymes	34.456	38.636
Monocalcium Phosphate	0.000	0.031
Salt	0.000	0.025

List of feed ingredients (g/100kg) of basal starter and grower diet for broiler chickens from day 0 to day 17 of age (day 15 to 17 are experimental days)

### ELISA assays

The ELISA assays for the detection of chicken APPs were obtained from Life Diagnostics Inc., (West Chester, PA, USA). The assays were performed according to the manufacturer’s instructions with specific dilution factors of 1:10000 for AGP (Catalog Number: AGP-5), 1:2000 for Ex-FABP ( Catalog Number: EXFABP-5) and 1:40000 for Hpx (Catalog Number: HPX-5). For SAA (Catalog Number: SAA-5) detection, protocol adjustments were made in the sample preparation due to the small amount of plasma recovered from the birds during the trials, 12.5 µl aliquots of plasma were used instead of the 100 µl recommended. Then, specific dilution factors were applied based on the time point: 1:20, 1:1280, 1:80, 1:20 at T0h, T12h, T24h, T48h respectively. Each sample well was measured at 450 nm using a FLUOstar Optima plate reader. The standard curve was generated using a four-parameter logistic curve (4PL) from the Optima software. The concentration of each APP was then calculated taking into account the dilution factor applied.

### Statistical analysis

GraphPad Prism v.9.1 was used for statistical analysis to explore the effects of diets (CTL, CTS and CMB) and LPS challenge at all time points pre and post challenge (T0h, T12h, T24h and T48h pre and post challenge) on AGP, SAA, Ex-FABP, Hpx abundance. When the difference between CTL, CTS and CMB was found not statistically significant, data from all dietary regimens per each APP were grouped to increase the sample size and to allow a clearer APPs comparison. A quantile-quantile (Q-Q) plot was use to check the normal distribution of data. All results were analysed using ANOVA with Tukey post-test analysis. Mean and standard error (SE) per each diet and all samples was specified. The statistical significance was set up at *P* < 0.05.

## Results

### Plasma concentration of AGP

Based on LPS challenge response in all dietary groups (CTs, CTS and CMB), AGP levels increased ∼6-fold, from 0.149 mg/ml pre challenge (T0h) mean to its peak of 0.919 mg/ml at T24h. A significant difference (*P* < 0.001) was discovered in all the time points post challenge (T12h, T24h and T48h) compared to T0h while no difference was found between T12h and T48h post challenge. No evidence of any dietary modulation of AGP was found based on CTS and CMB vs. CTL diet pre and post challenge. A lower AGP concentration was found in CMB diet compared to CTS at T24h (*P* < 0.05) (Table 2).

**Table 2:** AGP concentration pre (T0h) and post (T12h, T24h, T48h) LPS challenge in all dietary groups

Diet	T0h				T12h				T24h					T48h		
	**n**	Mean (mg/ml)	±	SE	**n**	Mean (mg/ml)	±	SE	**n**	Mean (mg/ml)	±	SE	**n**	Mean (mg/ml)	±	SE
CTL	12	0.130	±	0.012^**a**^	11	0.492	±	0.027^**b**^	11	0.874	±	0.061^**cd**^	11	0.459	±	0.037^**b**^
CTS	12	0.167	±	0.018^**a**^	12	0.509	±	0.019^**b**^	12	1.051	±	0.059^**c**^	12	0.576	±	0.035^**b**^
CMB	12	0.150	±	0.014^**a**^	12	0.454	±	0.021^**b**^	12	0.831	±	0.068^**d**^	12	0.462	±	0.049^**b**^
All samples	36	0.149	±	0.009	35	0.485	±	0.013	35	0.919	±	0.039	35	0.500	±	0.025

The table indicates the number of samples (n), the mean and standard error of the mean (SE). Means that do not share a letter are significantly different

### Plasma concentration of SAA

Plasma SAA concentrations increased dramatically at T12h post LPS challenge compared to all other time points (*P* < 0.001) (peak mean of 2.163 mg/ml) while no significant differences were observed based between broilers diets at this or all other time points (Table 3).

**Table 3:** SAA concentration pre (T0h) and post (T12h, T24h, T48h) LPS challenge in all dietary groups

Diet	T0h				T12h				T24h					T48h			
	**n**	Mean (mg/ml)	±	SE	**n**	Mean (mg/ml)	±	SE	**n**	Mean (mg/ml)	±	SE	**n**		Mean (mg/ml)	±	SE
CTL	12	0.0009	±	0.0002^**a**^	11	2.2830	±	0.1310^**b**^	11	0.0246	±	0.0046^**a**^	11		0.0016	±	0.0003^**a**^
CTS	12	0.0010	±	0.0002^**a**^	12	2.2680	±	0.1880^**b**^	12	0.0309	±	0.0056^**a**^	12		0.0017	±	0.0003^**a**^
CMB	12	0.0011	±	0.0001^**a**^	12	1.9490	±	0.1290^**b**^	12	0.0277	±	0.0048^**a**^	12		0.0019	±	0.0004^**a**^
All samples	36	0.0010	±	0.0001	35	2.1634	±	0.0898	35	0.0278	±	0.0029	35		0.0017	±	0.0002

The table indicates the number of samples (n), the mean and standard error of the mean (SE). Means that do not share a letter are significantly different

### Plasma concentration of Ex-FABP

In response to LPS, Ex-FABP was found to increase ∼40-fold, from 0.444 µg/ml mean at T0h, in all diets by peaking at T12h with 17.879 µg/ml mean (*P* < 0.001); then the level decreased at T48h (3.876 µg/ml mean) but was still ∼9-fold higher compared to T0h pre LPS challenge (*P* < 0.001). No difference in Ex-FABP concentration was observed comparing the CTL diet to each experimental diet (CTS and CMB) at any time point. A significant difference (*P* < 0.05) was found comparing the CTS vs. CMB diet at T12h and T24h; in both cases the CTS diet showed a higher concentration of Ex-FABP (Table 4).

**Table 4:** Ex-FABP concentration pre (T0h) and post (T12h, T24h, T48h) LPS challenge in all dietary groups

Diet	T0h				T12h				T24h					T48h			
	**n**	Mean (µg/ml)	±	SE	**n**	Mean (µg/ml)	±	SE	**n**	Mean (µg/ml)	±	SE	**n**		Mean (µg/ml)	±	SE
CTL	12	0.423	±	0.078^**f**^	11	17.169	±	0.566^**ab**^	11	13.325	±	0.397^**cd**^	11		3.594	±	0.464^**e**^
CTS	12	0.495	±	0.049^**f**^	12	19.725	±	0.805^**a**^	12	16.213	±	0.784^**bc**^	12		4.809	±	0.306^**e**^
CMB	12	0.413	±	0.100^**f**^	12	16.685	±	1.120^**b**^	12	11.993	±	1.020^**d**^	12		3.203	±	0.569^**e**^
All samples	36	0.444	±	0.044	35	17.879	±	0.541	35	13.858	±	0.540	35		3.876	±	0.283

The table indicates the number of samples (n), the mean and standard error of the mean (SE); means that do not share a letter are significantly different

### Plasma concentration of Hpx

LPS was found to significantly modulate the Hpx concentrations at T24h and T48h post LPS challenge compared to the physiological conditions pre challenge (T0h). Hpx was found to increase ∼5-fold and ∼4-fold at T24h (1429 µg/ml mean) and T48h (1163.9 µg/ml mean) compared to T0h (303.2 µg/ml mean) (*P* < 0.001). Based on dietary effects, no significant differences were found at any of the sampling time points (Table 5).

**Table 5:** Hpx concentration pre (T0h) and post (T12h, T24h, T48h) LPS challenge in all dietary groups

Diet	T0h				T12h				T24h					T48h			
	**n**	**Mean** (µg/ml)	**±**	**SE**	**n**	**Mean** (µg/ml)	**±**	**SE**	**n**	**Mean** (µg/ml)	**±**	**SE**	**n**		**Mean** (µg/ml)	**±**	**SE**
CTL	12	259.6	±	31.5^**e**^	11	578.0	±	52.8^**de**^	11	1263.6	±	61.4^**ab**^	11		986.7	±	96.2^**bcd**^
CTS	12	304.7	±	30.5^**e**^	12	667.1	±	53.3^**cde**^	12	1547.0	±	149^**a**^	12		1356.6	±	85.1^**ab**^
CMB	12	345.4	±	31.0^**e**^	12	654.9	±	44.8^**cde**^	12	1464.0	±	279^**ab**^	12		1134.0	±	149.1^**abc**^
All samples	36	303.2	±	18.4	35	634.9	±	29.0	35	1429.0	±	109.0	35		1163.9	±	69.2

The table indicates the number of samples (n), the mean and standard error of the mean (SE). Means that do not share a letter are significantly different

### APPs comparison

**Fig. 1:**
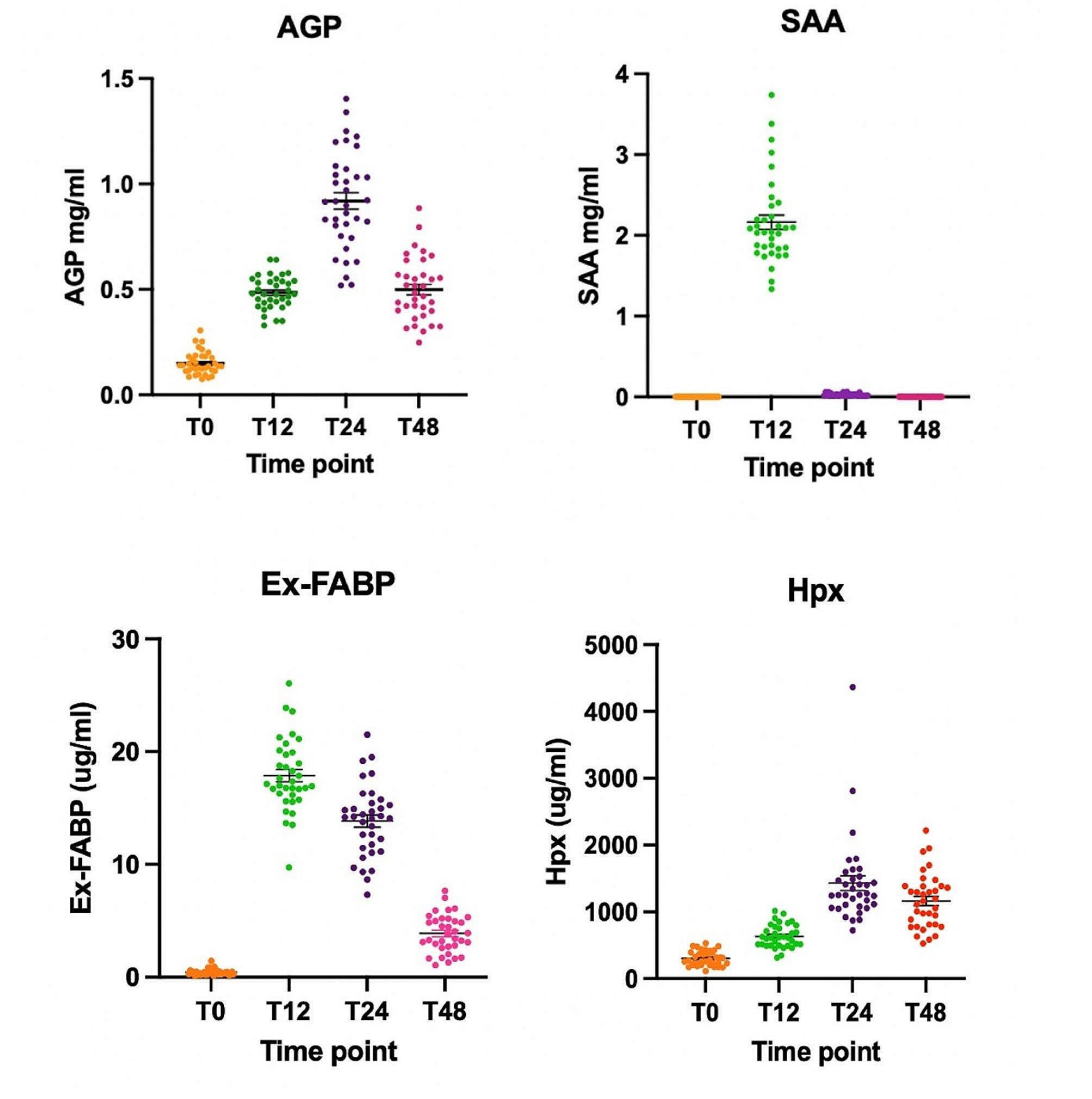
Scatter plots of AGP, SAA, Ex-FABP and Hpx concentrations pre (T0h) and post (T12h, T24h, T48h) challenge. All APPs positively reacted to LPS challenge with the strongest response (peak) at T24h for AGP, Hpx and T12h for SAA, Ex-FABP. Overall, Hpx showed a similar trend to AGP while Ex-FABP showed a similar trend to SAA

Dietary supplements, CTS and CMB, did not show significant effects of the modulation of established (SAA and AGP) and novel (Hpx and Ex-FABP) biomarkers. Based on this observation, the LPS challenge on these APPs was evaluated excluding the dietary variable to increase the sample size. Scatter plots for each APPs were generated to allow a better visualization of data for the APPs comparison (Fig. 1). The four APPs were confirmed to positively react to LPS challenge; AGP and Hpx showed a significant increase in their abundance at T24h post LPS challenge while SAA and Ex-FABP were signifiantly greater at T12h post LPS challenge (*P* < 0.001). The mean, peak and trend of each APP are also summarised in Table 6. Extracellular fatty acid binding protein (Ex-FABP) showed a similar early response to SAA post LPS challenge with an increase of ∼ 40-fold at T12h post challenge. However, the concentration in plasma and the measured APR response was more sustained for Ex-FABP than for SAA. Alpha-1-acid-glycoprotein (AGP) and Hpx showed a similar peak at T24h in response to LPS challenge with similar baseline concentrations, fold increase and trend.

**Table 6:** Comparison of novel and established APPs in plasma showing the mean plasma concentration of AGP, SAA, Ex-FABP and Hpx in samples from broilers subjected to a subcutaneous injection of *E. coli* LPS

APPs	Peak	Mean of APPs at the peak in all diets	Increased fold at the peak	APPs trend	APPs Restored after 48 h
AGP	T24h	0.92 mg/ml	∼6-fold	Slow increase and slow decrease	no
SAA	T12h	2.17 mg/ml	∼2000-fold	Rapid increase and rapid decrease	yes
Ex-FABP	T12h	0.02 mg/ml	∼40-fold	Rapid increase and slow decrease	not completely
Hpx	T24h	1.42 mg/ml	∼5-fold	Slow increase and slow decrease	no

The table indicates the LPS peak, concentrations at the peak, increased fold, trend and whether the concetration of each APP was restored to the level in healthy chicken at T48h after LPS injection

## Discussion

This study was designed to investigate and characterise the role of two novel APPs, Hpx and Ex-FABP, as APR biomarkers in broiler chickens challenged with *E.coli* LPS. Furthermore, the study investigated the anti-inflammatory activity of CTS and CMB dietary supplements by their ability to modulate the plasma concentration of established and novel APPs (SAA, AGP, Hpx and Ex-FABP) over a time course of 48 h pre and post challenge. To investigate these aims, the time course profile of the novel APPs, Hpx and Ex-FABP, responding to LPS challenge was compared to the more established APPs, SAA and AGP. In our study, SAA showed the greatest increase (∼2000-fold) in concentration post LPS reaching a peak at T12h. It then decreased rapidly at T24h to a level of ∼30-fold higher than at T0h and returned to the baseline normal physiological conditions at T48h. These results are in accordance with the existing literature [9, 10, 12] which suggests that measuring SAA at T12h post challenge provides a sensitive measure of the APR in broilers and is therefore classed as a major APP in this species. However, with the rapid return to the concentration found in healthy chicken in T24h, it could be useful in assessing the APP response in chicken to include SAA along with a panel of moderate more “long lived” APPs. This would provide additional insight into the temporal pattern of the APR as measuring SAA alone could lead to a key response event being missed by a failure to collect a sample at the appropriate time point. For example, Alpha-1-acid-glycoprotein (AGP) significantly increased to reach a peak at 24 h post LPS injection (∼6-fold) but its level started to increase (∼4-fold) in the first 12 h post LPS injection and by T48h it remained ∼4-fold higher than at T0h pre challenge. In chickens, AGP has been reported to increase of ∼5-fold or more in serum due to inflammation [11]. In a recent study, AGP concentration was found still significantly higher at T72h post LPS injection [12]. This would mean that AGP requires considerable time in broilers to return to its baseline physiological level. Our results, combined with these findings, confirm AGP as a moderate APR biomarker in broilers.

In terms of the two novel APPs described in the present study, Ex-FABP peaked at an increase of ∼40-fold in response to LPS at T12h; then its level decreased at T48h to ∼9-fold higher than the baseline level observed at T0h. Extracellular-fatty acid binding protein (Ex-FABP) has previously been reported to increase in plasma in response to inflammation and tissue degeneration in chickens [30]. Its mechanism of action as a component of the APR needs to be clarified, although it has been reported that Ex-FABP influences cartilage formation, muscle cell differentiation and heart development [31]. The expression of this protein increases in chicken’s jejunum with altered intestinal morphology after fasting or following bacterial infection suggesting that Ex-FABP may also play a role in stimulating cell proliferation, tissue repair and host defence [32]. A further study has also reported that Ex-FABP may have antibacterial activity via its ability to sequester iron binding siderophores during infections [33]. Based on these considerations, our results suggest that Ex-FABP may be used as a major APP biomarker of early inflammation in broilers.

Lipopolysaccharide was also found to significantly increase the Hpx concentrations ∼5-fold at T24h and ∼4-fold at T48h post challenge compared to the physiological conditions (T0h). This is in agreement with a study demonstrating that Hpx is a protein responsive to stress stimuli in chickens [9]. Hemopexin (Hpx) is a glycoprotein, mainly expressed in the liver tissue, whose synthesis is reported to increase ∼2 to5-fold as a result of inflammation [34]. Its functional role may be associated with a high binding affinity for heme, a highly toxic phosphoryl residue that can intercalate into lipid membranes inducing the production of free radicals [35]. The binding between heme and Hpx suggests that the role of Hpx is to act as a major vehicle of heme transportation in plasma thus preventing heme-mediated oxidative stress and heme-bound iron loss [35]. In mammals, haptoglobin (Hp) forms a complex with free plasma hemoglobin (Hb) to allow hepatic recycling of heme iron and prevent kidney damage [36]. However, in a previous proteomic study, the abundance of Hpx was found significantly greater than Hp in broilers post LPS challenge [12]. This may therefore indicate that in chickens, Hpx has a greater impact on heme preservation than Hp. Overall, the observed increase in Hpx during the LPS response in the current study supports the contention that Hpx may be a useful biomarker of inflammation.

In terms of dietary modulation, neither of the two dietary interventions (CTS and CMB) showed any consistent evidence of an immunomodulatory effect on the APPs tested at the given dose levels (CTS 300 g/ton; CMB 75 g/ton). However, a small but significant difference in AGP at T24h and Ex-FABP at T12h and T24h post challenge was detected when the CTS and CMB diets were directly compared suggesting that the latter may be worthy of further study, perhaps with a greater supplementation, as the CMB diet had a low but significant effect to reduce the APR compared to the CTS diet. The proposal that such dietary modulation could affect endotoxin activity in chicken has been supported by others studies [15, 24] that identified the ability of active compounds of the CTS or CMB diets to bind to bacterial LPS or alter the pattern recognition receptors (Toll-like receptors (TLR)) of macrophages, neutrophils, dendritic cells, and thereby attenuated the damaging effects of LPS. The available literature on the evidence of anti-inflammatory effects of CTS and CMB compounds in livestock may indicate that our experimental design related to factors such as the dosages of the CTS and CMB extracts and to the broiler age, could have limited the effects of the supplements on LPS treatment. Further investigations may consider different interventions such as greater extract dosages, different challenging models, other than *E.coli* LPS, later broiler’s age or experimental positive controls (i.e. broilers fed a diet supplemented with antibiotics). Based on these considerations, further investigation would be required to clarify the role of CTS and CMB diets in broilers pre and post challenge.

## Conclusion

Hemopexin (Hpx) and Extracellular fatty acid binding protein (Ex-FABP) were confirmed as potential biomarkers of APR in broilers when using an *E. coli* LPS model and they are of value along with SAA and AGP to fully characterise the chicken APR. However, no clear advantage of using either of dietary supplements to modulate the APR was identified at the dosage used. Future studies with greater extract dosage or other sources of stimulation of the APP response could be used to further investigate their potential value.

## Data Availability

The datasets used during the current study are available from the first author on reasonable request.

## Abbreviations

ABGPs: Antibiotic growth promoters
AGP: Alpha-1-acid-glycoprotein
APPs: Acute phase proteins
APR: Acute phase response
CMB: Cucumber diet
CTL: Control diet
CTS: Citrus diet
*E. coli*: *Escherichia coli*
Ex-FABP: Extracellular fatty acid binding protein
Hpx: Hemopexin
LPS: Lipopolysaccharide
Q-Q plot: Quantile-quantile plot
SAA: Serum amyloid A
SCFAs: Short chain fatty acids
